# Maturity Assessment of the Health Information System Using Stages of Continuous Improvement Methodology: Results From Serbia

**DOI:** 10.9745/GHSP-D-24-00083

**Published:** 2024-10-29

**Authors:** Steve Ollis, Milan Kovačević, Bosiljka Djikanovic, Nikola Radoman, Isidora Smigic, Mamadou Alimou Barry

**Affiliations:** a John Snow, Inc., Country Health Information Systems and Data Use, Arlington, VA, USA.; b Faculty of Medicine and Centre-School of Public Health, University of Belgrade, Serbia.; c Ministry of Health Republic of Serbia.; d National Alliance for Local Economic Development, Belgrade, Serbia.

## Abstract

**Introduction:**

Since the health information system (HIS) in public health care services in Serbia was introduced in 2009, it has gradually expanded. However, it is unclear how well the HIS components have developed and the whole system’s stage of maturity.

**Method:**

In June–September 2021, a maturity assessment of the Serbian HIS was conducted for the first time using the HIS Stages of Continuous Improvement (SOCI) toolkit. The toolkit measures HIS status across 5 HIS domains: leadership and governance, management and workforce, information and communication technology (ICT), standards and interoperability, and data quality and use. The domains were further divided into 13 components and 39 subcomponents whose maturity stage was assessed on a 5-point Likert scale, indicating the level of development: (1) emerging/ad hoc; (2) repeatable; (3) defined; (4) managed; and (5) optimized. The toolkit was applied in a working group of 32 professionals and experts who were engaged in developing the new national eHealth strategy and action plan.

**Results:**

The overall maturity score of the Serbian HIS was 1.6, which indicates a low level. The highest baseline score (2) was given to the standards and interoperability domain, and the lowest (1.1) was given to ICT infrastructure. The remaining 3 domains (leadership and governance, Management and Workforce, and Data Quality and Use) were similarly rated (1.7, 1.7, and 1.6, respectively).

**Conclusion:**

A baseline assessment of the maturity level of Serbian HIS indicates that the majority of components are between the emerging/ad hoc stage and repeatable, which represent isolated, ad hoc efforts, with some basic processes in place and existing and accessible policies. This exercise provided an opportunity to address identified weaknesses in the upcoming national eHealth strategy.

## Abstract (precis)

A structural and well-defined approach to the comprehensive assessment of the fragmented health information system points to items that could be improved in future governance efforts.

## Teaser

**Key Findings:**

- The Health Information System (HIS) Stages of Continuous Improvement (SOCI) toolkit was used to assess the maturity of the Serbian HIS across 5 domains using a 5-point Likert scale.
- The process of maturity assessment using the HIS SOCI toolkit in Serbia in 2021 served to establish a baseline status, strengthen dialogue among different key stakeholders in the field of digitalization of health care, and identify areas that were not covered by the initially drafted program for digitalization.

**Key Implications:**

- Policymakers should consider using available methods and tools for qualitative assessment of the maturity level of HIS before developing an eHealth strategy.
- The use of maturity assessment tools brings additional perspectives and considerations that could supplement and fortify the process of strategy development.
- Program managers can track the success of HIS development in the future using established and quantified baseline results of HIS maturity assessment.

## INTRODUCTION

The Republic of Serbia is a middle-income country in Southeastern Europe, commonly known as Western Balkan, with an assessed population size of approximately 6.6 million (without data for Kosovo and Metohija).^1^ The health care system in Serbia is organized and provided through a well-developed network of public health care services and institutions that function at the primary, secondary, and tertiary health care levels.^2^ Altogether, there are around 350 different entities (health care institutions) at the national level that are state owned and funded by the Republic Health Insurance Fund. Health information systems (HISs) have been largely implemented and used since 2009, according to the National Program of Work, Development, and Organization of an Integrated Health Information System – E-health, along with an action plan in 2009–2015, which provided a blueprint for the system development in later years.^3^

Since 2015, the HIS in Serbia has been incrementally developed and upgraded to enable some additional functionalities (e.g., electronic services) (Figure). For example, in 2016, general practitioners could make appointments at the secondary or tertiary level through the HIS. In 2019, eRecept (ePrescription) was introduced to allow paperless communication between primary care physicians and pharmacies to issue prescribed medicines to patients.^4^ However, despite these additional functionalities, key and strategic issues related to the HIS functioning, such as its governance and further development, remained unaddressed.

**FIGURE:**
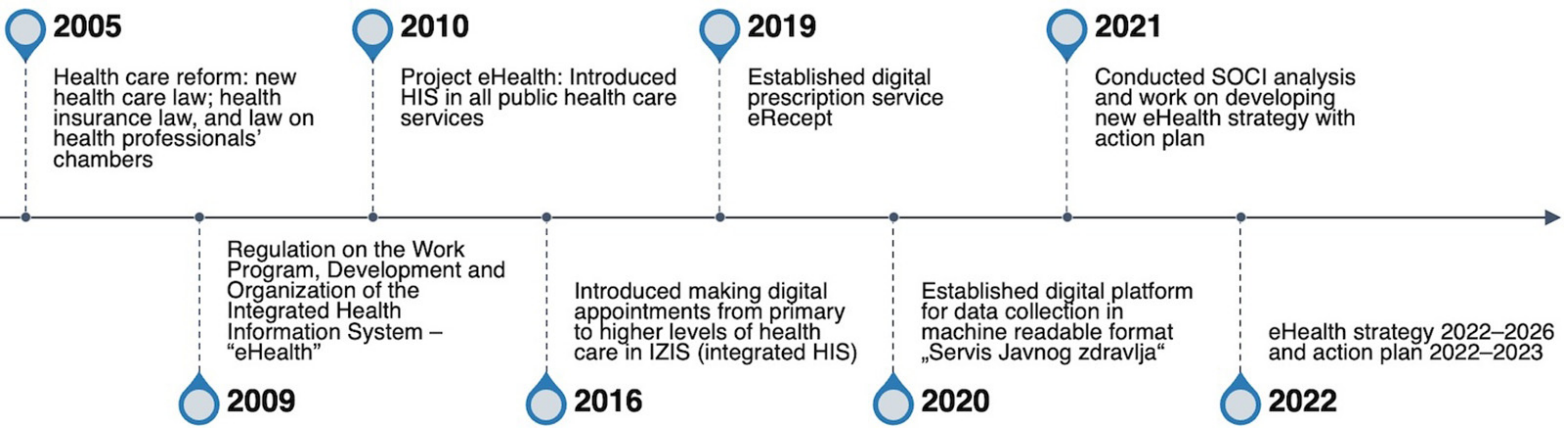
Timeline of Key Achievements in Developing the Serbia HIS Abbreviations: HIS, health information system; SOCI, Stages of Continuous Improvement.

The need to achieve a higher level of interoperability of the HIS in Serbia was recognized by the health system’s analysts, professionals, and officials in recent analytical reports in 2021 and the Prime Minister’s public address.^5^^,^^6^ Given the exponential growth of possibilities that information technologies (IT) and communications worldwide can provide, it was clear that the health care system in Serbia needed a strategic approach to further develop its HIS. The strategic approach required developing a national strategy and action plan that would provide a clear pathway for implementing new and innovative IT solutions in the health sector.

The need to achieve a higher level of interoperability of the HIS in Serbia was recognized.

To develop the national eHealth strategy and action plan, in 2021, the Prime Minister’s Office established the eHealth Steering Committee, a national task force co-chaired by both the Ministry of Health and the Prime Minister’s Office. The eHealth Steering Committee included 32 members from the ministries of finance, defense, and health, as well as representatives from various health institutions, health professional associations and chambers, and the private health care sector. The working group used the World Health Organization (WHO)/International Telecommunication Union National eHealth Strategy toolkit as a framework for developing the strategy and action plan.^7^

The development of the national eHealth strategy and action plan was largely supported by the Country Health Information Systems and Data Use (CHISU) program and other partners, including the United Nations Development Program.^8^ CHISU is a 5-year (2020–2025) U.S. Agency for International Development-funded initiative focused on strengthening HISs in more than 20 countries.^8^ CHISU operations in Serbia began in April 2021, with a mandate of strengthening the governance of the HIS and the use of health data for decision-making. During the process of developing the national eHealth strategy and action plan, CHISU was invited to participate and support the work of the eHealth Steering Committee.

### Assessment of the Health Information System Maturity Level in Serbia

To strengthen the foundation for developing the eHealth strategy and action plan and review the current maturity status of the Serbian HIS, an assessment toolkit called the HIS Stages of Continuous Improvement (SOCI) toolkit was used, as proposed by CHISU. The HIS SOCI toolkit had already been successfully applied in several low- and middle-income countries that intended to improve their HISs with donor support.^9^^–^^12^ The tool’s measurement scale evaluates processes, people, and functional and operational capabilities that facilitate collecting, managing, and using health information to support the health system.^13^ The HIS SOCI toolkit was developed by a team of HIS experts from MEASURE Evaluation, the U.S. Centers for Disease Control and Prevention, and the Health Data Collaborative digital health and interoperability technical working group.^13^ The SOCI toolkit provides a systematic process for assessing HIS maturity across 5 core domains: leadership and governance, management and workforce, information and communication technology (ICT), standards and interoperability, and data quality and use. These domains are divided into 13 components and 39 subcomponents (Table 1). Each subcomponent was assessed using the following 5-point Likert scale: (1) emerging/ad hoc; (2) repeatable; (3) defined; (4) managed; and (5) optimized, with 1 being the lowest and 5 the highest level of maturity. Descriptions of scores for each subcomponent are provided in Supplement 1,^13^ which was translated into the Serbian language to facilitate ease of use by all stakeholders.

**TABLE 1.** SOCI Baseline Assessment Scores^a^ for HIS Core Domains, Components, and Subcomponents, Serbia, 2021

**Core Domains**	**Average Score**	**Components**	**Average Score**	**Subcomponents**	**Points**
Leadership and governance	1.7	HIS strategic plan or HIS strategy	1.0	HIS strategic plan	1
				Monitoring and evaluation plan	1
		Policy, legal, and regulatory framework and compliance	2.0	Existence of HIS policies and legislation	2
				Policy compliance enforcement	2
		HIS leadership and governance organizational structures and functions	2.0	HIS leadership and coordination	2
				HIS organizational structure and functions	2
Management and workforce	1.6	HIS workforce capacity and development	1.3	HIS competencies (knowledge, skills, and abilities)	2
				HIS training and education (include continuous professional development)	1
				HR policy	1
		Financial management	2.0	HIS financing plan	2
				Resource mobilization	2
ICT infrastructure	1.2	Operations and maintenance	1.3	Reliable power/electricity	1
				ICT business infrastructure support	1
				Hardware	2
		Communication network (LAN and WAN)	1.0	Network and Internet connectivity	1
		Business continuity	1.0	Business continuity processes and policies	1
Standards and interoperability	2.0	Standards and guidelines	2.0	HIS standards and guidelines	2
				Data set definitions (clinical, laboratory, commodities, and indicator)	2
				Data exchange standards	2
		HIS core services	1.8	Master facility list	3
				Indicator registry	1
				Terminology management	2
				Unique person identity management	2
				Enterprise architecture	1
		Interoperability (data exchange)	2.3	Person data exchange	2
				Aggregate data exchange	2
				Community management data exchange	2
				Data security exchange	3
Data quality and use	1.4	Data quality assurance	2.0	Data quality assurance and quality control	2
				Data management	2
		Data use	1.2	Data use availability strategy	1
				Information/data availability	1
				Data use competencies	1
				User/stakeholder engagement	1
				Data synthesis and communication	2
				Reporting and analytics features	2
				Data use impact	1
				Data collection alignment with workflow	1
				Decision support (clinical or other)	1

Abbreviations: HIS, health information system; HR, human resources; ICT, information and communication technology; SOCI, Stages of Continuous Improvement.

^a^ Scores given using the following 5-point Likert scale: 1-emerging/ad hoc, 2-repeatable, 3-defined, 4-managed, and 5-optimized.

Conducting an HIS maturity assessment can lead to a thorough understanding of the HIS status, and the results can inform improvements in the HIS to a desired status by supporting the development of a clear roadmap to improve processes.

This article describes the results of the initial HIS SOCI assessment in Serbia as a baseline for comparison for future developments and an indication and reminder of areas that should be improved.

## METHODS

A SOCI assessment was conducted using a collaborative process in the following 5 consecutive steps.^13^

### Step 1. Establish the Assessment Core Team

The assessment core team was formed by several entities already engaged as part of the eHealth strategy and action plan Steering Committee. These included representatives from the Prime Minister’s Office, the Ministry of Health, the public health sector, the Institute of Public Health of Serbia Dr. Milan Jovanovic Batut (IPH Batut), and the National Alliance for Local Economic Development. These stakeholders from these entities were chosen based on their broad knowledge of the systems, processes, and policies related to the HIS. A complete list of the institutions from which assessment core team members came is given in Step 4.

### Step 2. Define the Scope and Assessment Approach

Before defining the scope and the assessment approach, multiple meetings took place to (1) present the concept of the assessment to the key high-level staff or leaders, (2) gain a better understanding of the SOCI toolkit and what needed to be done, and (3) select the best strategy to administer and conduct the SOCI assessment and receive key informant input.

To better understand the SOCI toolkit, the team discussed and addressed issues related to the SOCI terminology and assigning scores, the meaning of each score level, types of key documents to be reviewed to support the scores (evidence), and length of action planning and completing the SOCI assessment.

Given that the development of the eHealth strategy was already underway, the team decided that the best approach would be for the core assessment team to propose initial points per subcomponent, including citing available documents to justify the given points, where applicable, and present this to the broader working group for their review and discussion.

### Step 3. Conduct a Landscape Analysis and Desk Review

A thorough desk review and landscape analysis of relevant legislative and strategic documents and reports were performed to provide evidence on the situation in 2021 and justify proposed points per each subcomponent. Examples of reviewed documents included legislation (various laws and sublaws on health evidence and documentation, medical devices, patient rights, eBusiness, protection of personal data, public health, and protecting the population from infectious diseases); strategies that were relevant for HISs; official reports issued by public health institutions; and public procurement plans for Agencija za lekove i medicinska sredstva Srbije (ALIMS, Medicines and Medical Devices Agency of Serbia), Office for IT and eGovernment, and Health Insurance Fund.

At the eHealth Steering Committee’s request, CHISU provided experienced health experts who developed a summary of the current state of analysis, including all reviewed documents. The results were shared with stakeholders in a document summarizing the current state of eHealth legislation and policy in Serbia.

### Step 4. Selecting Key Stakeholders

The SOCI assessment was conducted as part of the eHealth strategy development process. As such, the stakeholders for SOCI were composed mostly of the eHealth steering committee members. The steering committee included 32 members who were representatives of the Prime Minister’s Office, the Ministry of Health, the Ministry of Defense, the Ministry of Finance, the Office for IT and eGovernment, the Republic Secretariat for Public Policies, IPH Batut, ALIMS, Medical Chamber of Serbia, Chamber of Commerce of Serbia, Pharmaceutical Chamber of Serbia, Chamber of Nurses and Health Technicians of Serbia, Association of Informatics in Health of Serbia, Association of Private Health Institutions and Private Practices of Serbia, Association of Patients of Serbia, the Council of Foreign Investors, the Nordic Business Alliance, the Digital Serbia Initiative, and the National Alliance for Local Economic Development.

The SOCI assessment was conducted as part of the eHealth strategy development process.

### Step 5. Organizing Assessment Workshops

The work on the development of the eHealth strategy and action plan was organized in 3 2-day workshops that were held in 2021. The sessions on the application of HIS SOCI toolkit were included in all 3 workshops.

At the first workshop, held on June 16 and 17, an overview of the SOCI toolkit was presented to the members of the eHealth Steering Committee, and a common understanding of the tool and its purpose was established. Following this workshop, the tool was translated into Serbian to improve its usability by all stakeholders.

At the second workshop, on August 11 and 12, the eHealth steering committee members broke into small groups and discussed the current state of the HIS across the domains and provided appropriate points based on scoring guidance. After this workshop, there was a follow-up with stakeholders to amend the desk review (Step 3) and obtain source documents to justify assigned points.

Between the second and third workshops, the HIS SOCI was emailed to the eHealth Steering Committee members so they could review the toolkit, propose points, and send it back with proposed points and comments to the assessment core team.

During the third workshop, on September 8 and 9, results from the assessment were presented and discussed with a wider group. During the discussion, consensus was achieved based on the evidence collected during the landscape analysis and desk review (Step 3) and arguments presented by stakeholders.

The outcome of the consensus method was a single point assigned to each of the 39 subcomponents on the following 5-point Likert scale: (1) emerging/ad hoc; (2) repeatable; (3) defined; (4) managed; and (5) optimized.

### Calculation of Scores

Scores for each of the 5 domains and 13 components within the domains were calculated as an average sum of points per component and per domain, presented to 1 decimal place.

## RESULTS

We identified 17 legislative and strategic documents, institutions, and digital platforms that were relevant for the HIS assessment according to the SOCI method. These were used to justify given points.^3^^,^^4^^,^^14^^–^^28^ Scores from an overall assessment of the maturity level for each of the 5 HIS core domains, components, and consensus-achieved points for the subcomponents are listed in Table 1.

### Leadership and Governance

The average score of 6 subcomponents in the leadership and governance domain was 1.7, which is closer to repeatable (2) than ad hoc/emerging status (1). Although Serbia has been making excellent progress in this area by announcing the development of the new eHealth strategy, the situation at the time of the assessment was rather modest because the last strategic document in this field was announced in 2009, with an action plan for the period 2009–2015.^3^ Some compliance documents and policies, such as the Law on Medical Documentation and Records in the Field of Health care, have not been updated to reflect the availability of digital systems,^18^ and an overall governing body for HIS development and supervision has not been defined yet. Key points in the areas of strength and major gaps and loopholes are presented in Table 2 for all domains.

**TABLE 2.** Summary of Main Findings in HIS SOCI Analysis in Serbia, 2021

**Domain**	**Areas of Strength**	**Major Gaps and Loopholes**
Leadership and governance	The Prime Minister’s Office recognized the need to define and adopt the eHealth Strategy. In January 2021, an eHealth Steering Committee was formed that was in charge of developing the eHealth Strategy and Action Plan. HIS policies and legislations are available to guide decisions and achievements of HIS outcomes in most areas/programs.^3^	The last strategy was announced in 2009, with activities from 2009–2015, so it has expired. Some key activities from that strategy have not yet been implemented, such as defining an organization/institution in charge of coordinating all aspects of eHealth. Lack of regularity in overseeing the function and implementation of the HIS and weak established process for sharing and reviewing HIS information with all HIS stakeholders.
Management and workforce	There are qualified personnel within Serbia, but they may not currently work in the public health system. For some positions and roles, requirements for IT staff are well defined in the national policy documents.^15^ National financing of HIS development within the last 10+ years has wisely used available international funds and credits, and almost all public health facilities currently have been equipped with HISs. Clearly defined rules for public financing.^16^^,^^17^	A lack of mechanisms for keeping and retaining qualified IT staff within the public health care system. There is no standard definition of the HR needs (the number and qualifications) for IT staff in health care facilities. A clear legislative framework for outsourcing IT services (i.e., regulations of the relationships between vendors [owners of operating IT systems] and health care facilities) is missing. There is no consolidated resource mobilization plan for further development and capital investments in HIS.
ICT infrastructure	Operations and maintenance of the IT systems are provided by vendors and their call centers. For centralized systems, support has been provided by MOH and IT Office. The majority of infrastructural components are in place, but operations, maintenance, redundancy, and security have to be improved. At the national level, there are robust Business Continuity Plans at the Office of IT and eGovernment and the National Data Center, which could support key system functions, such as data hosting and system developments.^25^	An overall and comprehensive system for technical monitoring of infrastructure, services, and central systems is missing. Central systems are not sufficiently resilient to respond to the temporary unavailability of local systems. Currently, local IT systems are not projected to provide a high level of targeted operability and availability (for example, 99% of the time) that is required for an integrated HIS. Monitoring and supervision of local IT systems is not provided. There is a lack of procedures to assure continuity in the work of local systems and disaster recovery.
Standards and interoperability	Use of already existing standards and guidelines have been established by normative regulations (legislation), primarily those related to infrastructure, public health, health insurance, and connecting subsystems in institutions.^14^^,^^18^^,^^19^^,^^21^ Existence of the portal eZdravlje (eHealth) for patients’ own monitoring of their health-related data and also scheduling appointments.^27^ Existence of the digital platform Servis javnog zdravlja (public health service) for official communication and data sets collection between institutes of public health and health care services.^26^ Interoperability between primary health care services and pharmacies is achieved through paperless, electronic prescription system eRecept (E-prescription), which is integrated into local HISs.^4^	Standards and guidelines for the HIS interoperability are not established yet; there is no centralized and coordinated system of their setting, application, localization, and control. Health care facilities at different levels are not able to exchange patient health records to provide a better quality of health care. Interoperability is low due to the lack of data exchange standards and common software solutions.
Data quality and use	The Institute of Public Health of Serbia defines and implements procedures for data collection, processing, analysis, and use at all levels of health care services in accordance with the national legislative obligations.^18^ Data might be available upon request, according to the Law on Free Access to Information of Public Importance.^28^ Working groups for developing and using HIS consist of representatives of all relevant stakeholders annual statistical yearbook is produced by the Institute of Public Health of Serbia.	Current data collection is not standardized, and data are not in a machine-readable format that allows for their synthesis and communication. There is no common, standardized format of data collection in a machine-readable format that would allow further data synthesis, report, and analysis, as well as quality assurance and control. There are no clear guidelines or procedures for access to depersonalized health data and their availability for further analysis. There is a lack of decision-making support tools based on updated and readily available registers of different kinds of machine-readable health-related data.

Abbreviations: HIS, health information system; HR, human resources; IT, information technology; MOH, Ministry of Health; SOCI, Stages of Continuous Improvement.

### Management and Workforce

The average score for the HIS management and workforce domain was 1.7. The HIS training and education (including continuous professional development) and human resources policy subcomponents received the lowest points because continuous professional development of IT staff and building and strengthening the knowledge and skills of IT solutions end users had been neither defined nor implemented and regulated. This indicated a clear need and window of opportunity for a systematic approach necessary for this improvement. In contrast, in Serbia’s health sector, there are well-defined and documented competencies, roles, and responsibilities for the workforce. Regarding the availability of the IT workforce with adequate HIS competencies, it was concluded and agreed that a knowledgeable and skillful IT workforce in Serbia exists, and therefore, the HIS competencies subcomponent (knowledge, skills, and abilities) received consensus-based 2 points. However, keeping them in the public health system is challenging because the globally competitive IT labor market offers better working conditions and higher salaries. Some national human resources policies regulate IT personnel positions and deployment, although they seem to underestimate the real needs for this type of staff.^15^

The subcomponents of HIS financing plan and resource mobilization were also assessed with 2 points because, in general, the use of public funds in Serbia is well defined, according to the Law on Public Procurement^16^ and the annual Public Procurement Plan of the Ministry of Health.^17^ These documents establish legal and administrative procedures for competition for public funds. However, regulations related to mobilizing resources and making investments in HIS that come from sustainable and ongoing resources have not yet been fully defined; therefore, the assigned point could not be higher in 2021.

### Information and Communication Technology Infrastructure

The HIS ICT infrastructure domain deals with the implementation of the required technology by applying standard operating procedures to enhance the daily business of the health sector and its stakeholders. The average score of the 5 subcomponents in this domain was 1.2. Only the hardware subcomponent through the consensus method was assessed with 2 points. The remaining subcomponents, reliable power/electricity, ICT business infrastructure support, network and Internet connectivity, and business continuity processes and policies, received 1 point (ad hoc/emergency status).

The average score of the 5 subcomponents in the ICT infrastructure domain was 1.2.

Despite these low points, some areas of strength exist, which are prerequisites for improvements (Table 2). The Office of IT and the independent eGovernment entity were established in 2017, and they provide national ICT infrastructure.^25^ The Office serves a variety of functions for the government and supports data hosting, systems development, and maintenance. In the future, it might have an expanded role in the development and maintenance of an HIS and support. Many eGovernment applications (e.g., the COVID-19 case tracking system) are hosted at the National Data Center. This key resource appears to be well staffed, with a high level of standardized processes in place. However, there was a lack of a unified vision and coordination between different ministries, which has resulted in a proliferation of systems and incomplete sharing of data and information between organizations/institutions.

Although backups of the system hosted at the National Data Center were being regularly conducted, when it came to the fragmented HIS, backups remained up to each health institution and their HIS providers (vendors) and were not done consistently. There is no central process for testing and monitoring the availability of the local systems, including network and system availability. This is especially important because the availability of patient data in the centralized system depends on the source system being online and available at the time of the data request.

### Standards and Interoperability

The HIS standards and interoperability domain and its 12 subcomponents are related to health data exchange between different entities and levels using nationally and internationally known and accepted standards. Compared to other domains, this was assessed the highest with an average score of 2.0, which meant that some procedures existed and were in a “repeatable” stage (Table 1).

Standards and interoperability of HIS in Serbia are assured by already existing standards and guidelines, primarily those related to infrastructure, public health, health insurance, and connecting subsystems in institutions, such as Regulation on the Detailed Content of Technological and Functional Requirements for the Establishment of an Integrated Health Information System; Law on Medical Documentation and Records in the Field of Health Care; and Professional Methodological Guidelines for Maintaining Basic Documentation on Resources in Health Care Institutions, Private Practice, and Other Legal Entities Engaged in Health care Activities.^14^^,^^18^^,^^19^ These documents’ existence and practical application justify the given points. However, there was also a recognized need to achieve a higher data standardization and interoperability level.

In the Serbian health care system, terminology management is assured by the use of the International Classification of Diseases and Related Health Problems version 10, which is integrated into the HIS.^21^ It enables Serbia to report selected health indicators to the official authorities in the European Union and the World Health Organization.^24^ At the national level, there is an e-service called Servis Javnog Zdravlja (public health service), which is hosted at the IPH Batut and allows aggregate data exchange between health care services and institutes of public health (i.e., the network of regional institutes, and IPH Batut).^20^^,^^24^^,^^26^ However, there are still many health-related datasets that ought to be collected as required by law^18^ and that are not reported through this service. There is much work ahead to be done to increase the interoperability of the systems and enable additional functionalities.

A good example of data exchange between primary health care physicians and pharmacies is eRecept.^3^ In addition, personal data exchange is provided by the Ministry of Health-hosted vertical health care management platform called eZdravlje (eHealth), which allows patients to monitor their health records, see the results of their health care visits, manage appointments, and perform other functions.^27^ Although this application and its outreach could be improved, it presents a good starting point for further development of the communication between citizens and health care services through digital platforms.

Unique person identity management is regulated by the existence and application of the Law on the Unified Register of Citizens and the Law on the Protection of Personal Data.^22^^,^^23^ However, this subcomponent could also be improved to ensure more data protection in the digital health care system.

### Data Quality and Use

The data quality and use domain with its 11 subcomponents indicates how well key stakeholders in the health system are using available data for decision-making and other purposes. The average score for data quality and use domain was 1.4. Four subcomponents, data quality assurance and quality control, data management, data synthesis and communication, and reporting and analytics features, were assessed with 2 points. The remaining 7 subcomponents were assessed with 1 point.

Procedures for data use and their quality are defined by the Law on Medical Documentation and Records in the Field of Health Care and corresponding Professional-Methodological Guidelines for Maintaining Basic Documentation on Resources in Health Care Institutions, Private Practice, and Other Legal Entities Engaged in Health Care activities, which are prepared by IPH Batut.^18^^,^^19^ They are implemented at all levels of health care within the national network of public health care institutions.^20^ Regional institutes for public health (24 of them) are in charge of data collection and control and send data to the IPH Batut for analyses at the national level.^20^ However, a functional national body or workgroup for quality management and data usage is needed with an established and standardized process that engages health data stakeholders. Moreover, Serbia has demonstrated the need for additional development of data quality plans that require periodic revisions using defined standards and procedures and methodological professional instructions at all levels. Strengthening the capacity of institutes of public health is crucial to continue to improve data collection and quality work.

## DISCUSSION

We present the results of the maturity assessment of the HIS in the Republic of Serbia in 2021. The assessment took a holistic approach and was done in a comprehensive and structured way that used a consultative process with members of a national working group that was in charge of developing the new eHealth strategy and applied the SOCI toolkit to assign consensus-based points to every subcomponent of the HIS across 5 domains.^13^^,^^28^ Several toolkits, such as the Digital Health Assessment Toolkit, Global Digital Health Index, and Maturity Model, have been used to assess the maturity of different HIS aspects, and they have been gradually evolving.^29^ In general, the results of these assessments serve to inform about the current state of HIS at the national level, clarify areas for further improvements, and serve as a baseline for comparisons with future progress.

The results of these assessments serve to inform about the current state of HIS at the national level, clarify areas for further improvements, and serve as a baseline for comparisons with future progress.

The implications of the HIS assessment in Serbia were beneficial for several reasons. It established a baseline for the maturity status in 2021, which can be used to monitor national progress in achieving desired goals in the future. This process also resulted in recommendations related to adding data quality and use as a program objective (a domain within the SOCI assessment that was missing in a draft plan), which was addressed in the national eHealth strategy.^30^ The assessment indicated that improvements were necessary for every component. The HIS SOCI toolkit was also used to set projected desirable points (goals) in each HIS subcomponent, which sum up the score of 2.9. Still, due to the limitations in the article length, we presented only the baseline assessment results. Projected goals are listed in Supplement 2.

HIS maturity assessments have been a widely recognized activity, which is especially beneficial for HIS analysis in low- and middle-income countries, such as Ethiopia, Kenya, Ghana, and Uganda.^9^^–^^12^ Improved use of digital technologies for health has been seen as a vehicle that can contribute to achieving universal health coverage, as stated by WHO in 2018.^31^ In addition, WHO’s Global Strategy on Digital Health 2020–2025 emphasized that an integral part of health priorities should be digital health, and populations should benefit from it “in a way that is ethical, safe, secure, reliable, equitable and sustainable.”^32^

The comprehensive HIS maturity assessment results clearly indicated where the sector was in terms of 5 key domains. Overall, it showed that the average national HIS maturity level across all 5 domains was 1.6, between emerging/ad hoc and repeatable. This score means there was an awareness of the need to develop different components of HIS, but ad hoc solutions and fragmented approaches dominated. Project-based activities were not sufficiently coordinated among all stakeholders, and there was insufficient institutional framework and support. The assessment also revealed that there was a clear need for standardization of the data collection and data exchange in a machine-readable form, in addition to the need to create an entity/body that would coordinate and oversee the implementation of the program for digitalization in the health system in Serbia and action plan.

The average score for the ICT infrastructure domain was lower (1.2) than the domain HIS standards and interoperability (2.0), which may be surprising considering that the infrastructure is a prerequisite for HIS standards and interoperability. However, we believe that the discrepancy in these scores reflects the level of key informants’ subjective satisfaction with the basic infrastructure, experiencing temporary failures of HIS or lack of IT supplies in a few health facilities. The SOCI tool did not propose quantifying infrastructure; its scores were based on the description, existence of the relevant documents, and mutual consensus, not on quantification, so it is difficult to assess it objectively and compare these results with assessments conducted in other countries worldwide, which we hesitate to do.^9^^–^^12^ The level of satisfaction with the current state of availability of HIS standards and interoperability could be ascribed to recently adopted new system’s functionalities and interoperability, such as a digital platform for data collection Servis Javno zdravlje^26^ or (eRecept).^4^

In May 2022, the eHealth strategy and action plan was adopted,^33^ and all important elements of developing an integrated HIS in Serbia are envisaged and broken down into concrete objectives, measures, and activities. The level of its implementation remains to be seen (i.e., the level of achieving desirable improvements in the upcoming period).

### Limitations

We acknowledge that the SOCI tool applied in this research had limitations that were beyond our control. Specifically, the descriptions of the points assigned to subcomponents within the domains were broadly defined, which may have led to variations in individual interpretations depending on the individual perspective, experience, and perceptions of the key informants most involved in the digitalization of the health system. However, final assessments were formed by mutual (dis)agreements, discussions, and eventually achieving consensus, though we acknowledge that this process may have been subject to bias. In some cases, different perspectives among steering committee members led to varied interpretations of the level of maturity development, with those who articulated their views more effectively influencing the group’s consensus. As a result, it is challenging to determine whether all members fully embraced the perspective that reached a consensus or if they agreed to avoid further discussion. An alternative approach to the consensus method could have been the Delphi method, where participants independently assign scores over several rounds without interacting with each other.^34^ However, we felt that this method might not foster mutual collaboration, exchange of opinions, and capacity-building within the country. For these reasons, we chose to proceed with the consensus method.

The SOCI tool is, by default, meant for systematically evaluating the overall national HIS and its governance, not its fragments, and we acknowledge that end users might have different opinions and perspectives over the level of HIS maturity.

By applying the SOCI tool, we intended to provide a wide landscape of the domains pertinent to digitalization, but they were defined and limited by the proposed SOCI method. Indeed, we recognized that not all important fields were addressed and evaluated by this tool, such as the applicability of artificial intelligence (AI) in health care. Nevertheless, the use of AI was addressed in the eHealth strategy and action plan,^33^ which indicates a high level of awareness at the national level in Serbia regarding the opportunities of IT and HIS. In the Action Plan, AI was mentioned within activities, such as the regulation of the legal framework for AI application in the health care system; the use of AI in radiological diagnostics, real-time drug interaction checks during the creation of electronic prescriptions, embedding best practice guides and clinical pathways into software solutions, and a range of other highly sophisticated IT solutions for improving clinical practice.^33^

## CONCLUSION

The 2021 SOCI assessment indicated that the level of HIS development in the Republic of Serbia was somewhat low. Still, it provided a good foundation for the activities planned in the health sector. The newly created and adopted eHealth strategy and action plan provide strategic directions and a blueprint for further development of an integrated HIS.^33^ This integrated HIS would allow a higher level of interoperability, quality, and use of health-related data in circumstances that are characterized by a high level of governmental commitment to defining an appropriate institutional and legislative framework that would be required for further HIS development.

## Supplementary Material

24-00083-Djikanovic-Supplement_2.xlsx

24-00083-Djikanovic-Supplement_1.xlsx

## Notes

**Peer Reviewed**

**First Published Online:** October 17, 2024.
